# Development and validation of the Australian Aboriginal racial identity and self-esteem survey for 8–12 year old children (IRISE_C)

**DOI:** 10.1186/s12939-015-0234-3

**Published:** 2015-10-24

**Authors:** CS Kickett-Tucker, D. Christensen, D. Lawrence, SR Zubrick, DJ Johnson, F. Stanley

**Affiliations:** Australian Catholic University & Pindi Pindi, Centre for Research Excellence in Aboriginal Wellbeing, 20 William Street, Midland, WA 6935 Australia; Telethon Kids Institute, The University of Western Australia, 100 Roberts Road Subiaco, Western Australia, 6008 Australia; Michigan State University, 552 W. Circle Drive, East Lansing, MI 48824 USA

**Keywords:** Instrument development, Racial identity, Self-esteem, Australian Aboriginal children

## Abstract

**Introduction:**

In Australia, there is little empirical research of the racial identity of Indigenous children and youth as the majority of the current literature focuses on adults. Furthermore, there are no instruments developed with cultural appropriateness when exploring the identity and self-esteem of the Australian Aboriginal population, especially children. The IRISE_C (Racial Identity and Self-Esteem of children) inventory was developed to explore the elements of racial identity and self-esteem of urban, rural and regional Aboriginal children. This paper describes the development and validation of the IRISE_C instrument with over 250 Aboriginal children aged 8 to 12 years.

**Methods:**

A pilot of the IRISE C instrument was combined with individual interviews and was undertaken with 35 urban Aboriginal children aged 8–12 years. An exploratory factor analysis was performed to refine the survey and reduce redundant items in readiness for the main study. In the main study, the IRISE C was employed to 229 Aboriginal children aged 6–13 years across three sites (rural, regional and urban) in Western Australia. An exploratory factor analysis using Principal axis factoring was used to assess the fit of items and survey structure. A confirmatory factor analysis was then employed using LISREL (diagonally weighted least squares) to assess factor structures across domains. Internal consistency and reliability of subscales were assessed using Cronbach’s co-efficient alpha.

**Results:**

The pilot testing identified two key concepts - children’s knowledge of issues related to their racial identity, and the importance, or salience, that they attach to these issues. In the main study, factor analyses showed two clear factors relating to: Aboriginal culture and traditions; and a sense of belonging to an Aboriginal community. Principal Axis Factoring of the Knowledge items supported a 2-factor solution, which explained 38.7 % of variance. Factor One (Aboriginal culture) had a Cronbach’s alpha of 0.835; Factor 2 (racial identity) had a Cronbach’s alpha of 0.800, thus demonstrating high internal reliability of the scales.

**Conclusion:**

The IRISE_C has been shown to be a valid instrument useful of exploring the development of racial identity of Australian Aboriginal children across the 8–12 year old age range and across urban, rural and regional geographical locations.

## Introduction

It is generally accepted that having a positive view of oneself is beneficial to health and wellbeing. Those with a high self-esteem (or self-concept) can cope more efficiently with life's challenges; they feel valued, respected and generally lead happy and productive lives [1]. Those “who feel good about themselves and their abilities are likely to be more effective than individuals with low self-concepts” and are less likely to have anxiety or depression [2].

Links between racial identity (also referred to as cultural identity, ethnic identity, or racial-ethnic identity) and positive self-esteem have been explored by various researchers around the world. Corenblum [3] states that “racial-ethnic identity and self-esteem are important indicators of positive mental health and adjustment among low status and minority group members”, and that positive racial identity provides a buffer against the negative impacts of prejudice and discrimination often experienced by minority groups. In general, the extent to which one’s cultural group is recognised and clearly defined in one’s mind is positively related to a clear definition of one’s self and subsequently, one’s self-esteem [4–6].

Since the 1960’s, researchers have developed measures of self-esteem. These encompass relatively long scales, such as the *Piers-Harris Children’s Self-Concept Scale* [7] and the *Coopersmith Self-Esteem Inventory* [8] as well as the shorter *Rosenberg Self-Esteem Scale* [9] originally designed to measure self-esteem in high school students. In Australia, Marsh [10] developed a *Self-Description Survey* which measures nine factors specific to physical self-concept: Activity, Appearance, Body Fat, Coordination, Endurance, Flexibility, Health, Sport and Strength, as well as measures of Global Physical and Global Esteem.

Other researchers have moved away from generic measures of self-esteem to measures more explicitly centered on racial and cultural identity. The “Multi-dimensional Model of Māori Identity and Cultural Engagement” [11] was designed specific for Māori populations in New Zealand. It was developed to measure identity and cultural engagement, but does not specifically focus on the link between racial identity and self-esteem. It was also designed and tested on adults aged 18–74 years.

The link however between self-esteem and racial identity for Australian Aboriginal children has never been made or rarely estimated. Historically, health and wellbeing frameworks have been developed using research based on general population groups. Many of the concepts in existing scales do not align themselves with Aboriginal worldview and particularly concepts of health and wellbeing. For Australian Aboriginal populations, the definition of health “is not just the physical wellbeing of an individual, but the social, emotional and cultural wellbeing of the whole community” [12]. Hence, it is important to develop instruments specifically for the Australian Aboriginal experience, with Aboriginal involvement in all stages of research. This is “a matter of both ethics and rigor and … is considered vital if health inequalities are to be addressed” [13]. Furthermore, any programs developed from the findings of studies using the instrument are unlikely to be effective if there is not a “high level of Indigenous ownership and community support” [14].

The measurement of self-esteem across cultures can be difficult, particularly when one culture places a higher value on particular issues than others. For example, Western cultures tend to place a higher value on issues of independence and self-reliance, whereas more traditional societies (such as Aboriginal groups) more commonly value collectivism and a strong reliance on social support [15]. School achievement is a good example. Self-esteem might be more valued in Western society where the emphasis is less on family and community extensions of self but on individualism and competition [15].

A measure of self-esteem developed in Western cultures will not necessarily be valid in other societies. Purdie & McCrindle [15] discuss a range of potential sources of error that can occur when using an instrument developed in a different culture. Firstly, concepts may be interpreted differently across cultures. Secondly, there may be a degree of method bias introduced, in terms of how the instrument is administered and whether it is culturally appropriate. Response errors increase when the instrument is designed and administered by a person from a different culture [16]. Responses could be affected by reading ability (which may not be particularly strong in some cultures), by expected cultural norms (some societies are known to always answer in the affirmative, so as not to disagree with the interviewer) and by the race of the interviewer (if different to the respondent) [15]. Finally, item bias may occur if questions can have different meanings for different cultural groups, for example, the use of colloquialisms may affect a respondent’s understanding [15].

Aside from needing an instrument that is specifically designed for Aboriginal populations, the instrument needs to be suitable for children and use language that Aboriginal children will relate to. Racial identity is formed gradually, starting from as young as 4 years of age and developing more fully as a child grows. Byrd [17] identifies three aspects of racial identity: a) awareness (the ability to distinguish between members of different races); b) identification (the ability to name one’s own race); and c) attitudes (beliefs about the characteristics between different racial groups). Awareness and identification become stable by adolescence, so studies that deal with racial issues in adults focus mainly on attitudes. However, in children awareness and identification are also important. The age at which awareness develops varies, but most children are not able to correctly classify individuals by race until they are four or five, although awareness begins as soon as they can notice differences in people’s skin colour, hair, eye colour, which can be distinguished as young as 2–3 years old [18, 19]. Ability to self-identify comes by around four to six years of age. Knowing that one’s race will not change (racial constancy) develops somewhat later, at around ten to twelve years. Children gradually increase the complexity of their understanding of racial differences as they grow older.

It is important to be able to track how racial identity and self-esteem develop over childhood and adolescent years. Currently, most available tools for measuring self-esteem have been developed for adults or adolescents (with some modified for children) and lack specificity to Aboriginal populations. A self-report survey is not best suited to younger children who are still developing literacy skills. We report here on a self-esteem scale called the IRISE-C, designed by an Aboriginal researcher based on a literature review and Aboriginal community consultation, and using terms that would be well understood by Aboriginal children. It was administered by Aboriginal Community Research Assistants (ACRA) asking and recording the children’s answers, eliminating the need for literacy proficiency. The terminology used in the IRISE_C mirrors the language Aboriginal children use and thereby reducing misunderstanding of questions. Aboriginal Community Research Assistants asked each question and used visual score charts for the participants to indicate their responses. The purpose, content and procedures for the IRISE_C have been developed in accordance with the wider Aboriginal community’s communication protocols and culturally safe and secure practices deemed appropriate for Australian Aboriginal children aged 8–12 years.

## Methods

### Design

This research employed a mixed methods design to allow the pilot and main study procedures and findings to support and complement each other. In the pilot study, data were gathered from several sources including in-depth personal interviews, surveys and students’ school reports. For the main study, data were collected from surveys.

Ethical approval was obtained from the Human Research Ethics Committee at Murdoch University (Approval 2009/163), Western Australian Health Ethics Committee (Approval 244 06/09) and the Western Australian Department of Education (Approval D10/0358138).

### Pilot study

The IRISE_C was employed in a pilot study whereby the instrument consisted of 71 items and explored the following themes (subscales): 1. Knowledge and/or experience of identity; 2. Salience placed upon identity; 3. Knowledge and/or experience of culture and; 4. Salience placed upon culture. The IRISE_C utilised a four point Likert scale with subjective responses ranging from ‘none’, ‘a little bit’, ‘some’ and ‘a lot.’ The purpose of the pilot study was to trial the IRISE_C inventory with a larger sample across the expected age range of 8–12 year olds. The objective of the pilot study was to gather preliminary data to help guide the main study.

#### Recruitment of participants

In the pilot study, the participants consisted of 35 urban Aboriginal children aged 8 to 12 years old attending co-educational State Primary schools in metropolitan Perth, Western Australia. Five schools were randomly selected. Individual participants were identified as Aboriginal using information supplied on the school enrolment form. This form was completed by carers upon their child’s enrolment at the commencement of the school year. Carers voluntarily reported their child’s Aboriginal identity. Of the total number of participants, 16 were male (45.7 %) and 19 were female (54.2 %). Pilot study participants were in grades ranging from 2 to 7, with the majority in grades 5 and 6.

#### Administration

A combination of survey and taped interviews were conducted with each Aboriginal child and were administered by trained Aboriginal Community Research Assistants at the school site. Telephone surveys were then conducted with the primary carer for each child. This process was necessary to collect family demographic information. Other data sources collected included student academic reports, behaviour reports and student profiles, which were provided by the respective schools.

#### Procedure

The IRISE_C is a paper survey which was verbally administered one on one to school children by a trained, local Aboriginal Community Research Assistant (ACRA). The scoring key was placed in front of the child participant. The scoring key is A4 in size and contains the Likert scale with images of “smiley faces.” The pre-pilot and subsequent pilot of the scoring key revealed that school children, who are the target group were very familiar with the use of smiley faces on the IRISE_C scoring chart as it was an acceptable, recognisable symbol.

Aboriginal Community Research Assistants asked each child to read the scoring chart so that they could assess if the child: 1. was able to read the words; 2. understood what the words meant; 3. was familiar with the scale even if they could not read the words; and 4. were confident and comfortable in making a decision. This step was repeated until the ACRA made an assessment that each child could without reservation (i.e., without stalling, without taking more than 5 s to respond to exercise questions, non-response, looking away, or looking directly at the ACRA for more than 5 s) provide a verbal answer or indicate their response by pointing to the answer on the IRISE_C Scoring chart. In the pilot study, no child was turned away from participation because of the assessment made by the ACRA. Young children (8–9 years) were more likely to point to the chart, whereas older children (10–12 years) provided a verbal response to survey items. After reading each question to the participant, the ACRA recorded the child’s response by marking the appropriate answer on the survey form.

An SPSS [20] database was developed and all individual responses were recorded accordingly. The database also included secondary data such as school attendance records, achievement, school behavior records and school health records.

#### Scale revision

The pilot data were used to refine the items contained in the survey. An exploratory factor analysis was undertaken to identify redundant items, items with little or no variability, and items that had minimal relationship to the underlying concepts of racial identity and self-esteem. This resulted in the 71 items used in the pilot version of the scale being reduced to 40 items. The 40-item version was then employed in the main study.

### Main study

#### Acceptability of instrument

Consisted with the principles, values and ethics for maintaining cultural security when working with Australian Aboriginal communities [21], selected Aboriginal community members and professionals working with Aboriginal children were invited to assess the acceptability of the IRISE_C instrument.

Consequently, this group of individuals deemed the IRISE_C an acceptable survey in which to capture the elements of Aboriginal identity and related self-esteem which is evidenced in their contributions during stages 3 and 4 of instrument development and their participation during the dissemination of results and of which, the carers and parents of children also contributed. Further evidence of the instrument acceptability is provided in the response rates for the pilot and main study which were 70 % and 91.7 % respectively. These numbers reveal parents’ and carers’ acceptability in allowing their children to participate. No issues were reported during the study in relation to acceptability or use of the survey.

#### Use of instrument

Local Aboriginal protocols were observed when employing the IRISE_C instrument. More specifically, being respectful of each individual Aboriginal child and showing that the interviewer is genuine (using appropriate verbal and non-verbal language) are key ingredients in growing rapport and eventually a ‘working relationship’ with Aboriginal children. These elements are vital to the success of this project in achieving authentic and reliable data and these were maintained by:

- Recruiting Aboriginal Community research assistants from the local area of the study site and training them in interviewing and survey techniques.
- Surveys being administered on a one-on-one basis with each participant.
- Verbally asking each individual survey item of participants and recording the response accordingly on the paper survey
- Using a visual (image) score card.

Scientific protocols were also observed and maintained (underpinned by Aboriginal community protocols) and these were very important to ensuring children’s understanding of survey items:

- Provision of 2 practice questions at the commencement of the survey.
- Placing a visual score card in front of the child in which they used to indicate, by pointing or verbal communication of their answers.
- ACRA observed the participant’s eyes as they tracked the visual score card for their responses, thus demonstrating their willingness to seek an answer to items.
- Reading body language and taking note of verbal cues help to ascertain any difficulties as well as the level of engagement of participants during the survey. Key characteristics that were recognisable when participants were not engaged or had difficulty were: a) gazing elsewhere, b) slouching, c) head down, d) fidgety, e) blank look, f) silent or shrugging shoulders when asked a question, g) responding with “what?” when asked a question and h) general inattentiveness.

#### Recruitment

The sample was recruited from 28 schools located in 3 locations including rural (Goldfields in Goldfield district), regional, (Peel in south metro district) and metropolitan (Swan in north metro district).

Most participants attended co-educational State Primary schools however; one independent school was included in this study. Schools were randomly selected and 10 accounted for Swan schools, 16 in Peel and a further 4 schools in the Goldfields. Similarly to the pilot study, individual participants were identified as Aboriginal using information supplied on the school enrolment form.

#### Participants

Participants in the main study consisted of 229 children aged 6 to 13 years of age (mean age, 10 years), and year 3 to 7 (mean = year 5) (Table 1).

**Table 1:** Sample characteristics

Gender					
		Frequency	Percent	Male	Female
Age	6	1	.4	0	1
	7	8	3.5	4	4
	8	35	15.3	13	22
	9	47	20.5	22	24
	10	45	19.7	24	19
	11	46	20.1	25	20
	12	38	16.6	19	19
	13	7	3.1	4	3
	Missing	2	.9		
School grade	3	41	17.9		
	4	49	21.4		
	5	44	19.2		
	6	52	22.7		
	7	37	16.2		
	Missing	6	2.6		
Major Aboriginal or Torres Strait Islander Group	Noongar	110	48.0		
	Wongi	28	12.2		
	Yamatji	16	7.0		
	Koori	4	1.7		
	Gooniyanati	1	.4		
	Yeroo	2	.9		
	Pulku	2	.9		
	Torres Strait Islander	1	.4		
	Missing	65	28.4		
Non-Aboriginal Heritage	Yes	83	36.2		
	No	91	39.7		
	Don’t know	24	10.4		
	Sometimes	1	.4		
	Missing	30	13.1		

#### Administration

The IRISE_C survey was verbally administered to children by the Aboriginal Community Research Assistants (ACRA). A vital element of the main study was the selection and recruitment of ACRAs. Successful selection was based on ACRA’s knowledge of family networks, access to schools and demonstrated rapport with children. Participants’ responses to individual survey items were recorded by the ACRA on paper forms and subsequently entered onto a SPSS database [20].

#### Procedure

The items were tested on 229 children from the Swan (*n* = 87), Peel (*n* = 71) and Kalgoorlie (*n* = 71) regions of Western Australia. Two hundred and twenty seven (227) children acknowledged some form of Aboriginal identity, with the other 2 children having missing data. Children also varied in their rate of acknowledgement of non-Aboriginal heritage. Children were able to indicate their Aboriginal group name, with 164 children indicating at least 1 group identity, 47 indicating membership of at least 2 groups, and 6 children indicating membership of 3 groups. Response rates to IRISE-C questions varied (*n* = 210–226), giving a ratio of approximately 6 children per item. Although guidance on adequate sample for exploratory factor analysis varies, Costello and Osborne [22] would deem this an adequate sample for confirmatory factor analysis.

#### Methods of analysis

The fit of items and structure of the survey was first assessed using Exploratory Factor Analysis and then validated using Confirmatory Factor Analysis. The exploratory factor analysis was undertaken in SPSS [20]. Principal axis factoring was used, with an oblique (promax) rotation, as there was no a priori basis for assuming that different aspects of racial identity and self-esteem would be independent. The internal consistency and reliability of each sub-scale was assessed using Cronbach’s co-efficient α.

Following identification of a similar factor structure in both the knowledge and salience items of the IRISE_C, a confirmatory factor analysis was used to assess the fit of a consistent structure across both knowledge and salience domains. The confirmatory factor analysis was undertaken using LISREL, using the method of diagonally weighted least squares.

## Results

### Exploratory factor analysis

#### Knowledge questions

A visual inspection of the correlations matrix for the knowledge questions identified several correlations above 0.3 in the dataset, with each item (other than Q.26. ‘How much do you get ‘shame’ because you are Aboriginal?’) having correlations above 0.3 with other items. The KMO was 0.873 and Bartlett’s test of sphericity was significant (chi-square (171) = 1127.3, *p* < 0.001), supporting factorability of the dataset.

In the analysis 2, 3 and 4 factor solutions were examined, but the 2-factor solution came closest to simple structure. An initial analysis showed all items had communalities greater than 0.2, other than Q.21. ‘Like to have a good laugh’ and Q.26. ‘How much do you get ‘shame’ because you are Aboriginal’ (reverse coded). After consultation with the survey creator, these items were excluded from this analysis as field experience indicated that the children in the study did not understand these concepts which may better apply to the racial identities of older children.

Principal Axis Factoring of the Knowledge items supported a 2-factor solution, which explained 38.7 % of variance. The correlation between the knowledge factors was 0.628. The first factor represents ‘Aboriginal culture,’ the second factor represents ‘racial identity’. Although not shown, there was also evidence to support combining all measures into an overall ‘omnibus’ measure.

Factor One (Aboriginal culture) had a Cronbach’s alpha of 0.835; Factor 2 (racial identity) had a Cronbach’s alpha of 0.800 (Table 2).

**Table 2:** Knowledge items (restricted item pool, excludes ‘shame’ and ‘laugh’)

Item	Factor Loading — Factor 1 Aboriginal culture	Factor Loading — Factor 2 racial identity	Item variance explained by factors one and two
Q1. How much do you know about how Aboriginal people lived in the old days?	0.544		0.253
Q3. How much do you know about Aboriginal Week activities?	0.398		0.169
Q5. How much do you like playing with Aboriginal kids?		0.652	0.447
Q7. How much have you learned to make Aboriginal foods like damper?	0.739		0.415
Q9. How much do you like being Aboriginal?		0.746	0.464
Q11. How much are you the same as other Aboriginal kids?		0.374	0.234
Q13. How much do you know about Aboriginal stories of the Dreaming (Dreamtime)?	0.547		0.340
Q15. How much do Aboriginal kids make you feel part of their group at school?		0.393	0.340
Q17. How much do you talk Aboriginal words?	0.594		0.375
Q19. How much does your family tell you about being Aboriginal?	0.604		0.431
Q23. How much do you like Aboriginal people as friends?		0.479	0.330
Q28. How much are you proud of being Aboriginal?		0.93	0.737
Q30. How much do Aboriginal kids help each other?		0.339	0.315
Q33. How much do you like the Aboriginal flag?		0.636	0.393
Q35. How much do you go bush with your family?	0.540		0.332
Q37. How much have you eaten Aboriginal foods, like kangaroo?	0.546		0.446
Q39. How much do you know about the dances Aboriginal people did in the old days?	0.700		0.554
Total variance explained (%)	32.3 %	6.3 %	
Cronbach’s alpha	0.835	0.800	
Correlation between factors 1& 2 (knowledge): 0.628			

#### Salience questions

A visual inspection of the correlations matrix identified that with each item had correlations above 0.3 with other items. The KMO was 0.913 and Bartlett’s test of sphericity was significant (chi-square (136) = 1242.85, *p* < 0.001), supporting factorability of the dataset.

Following the analysis of the questions relating to knowledge, the salience questions relating to having a laugh (Q.22. ‘How important is it to you to have a good laugh’) and shame (Q.27. ‘How important is it for you to not be ‘shame’ of being Aboriginal’) were excluded from the analysis.

In the analysis, 2, 3 and 4 factor solutions were examined, but the 2-factor solution came closest to simple structure. An initial analysis showed all items had communalities greater than 0.2.

Principal Axis Factoring of the salience items supported a 2-factor solution, which explained 44.6 % of variance. The correlation between the salience factors was 0.691. Only one item (Q. 38 How important is it for you to eat Aboriginal foods?) cross-loaded. The pattern of loadings was reversed relative to the knowledge items, with the second factor extracted representing ‘Aboriginal culture’, and the first factor extracted the second factor representing ‘racial identity’ (Table 3).

**Table 3:** Salience items (complete item pool, excludes ‘shame’ and ‘laugh’)

Item	Factor Loading — Factor 2 Aboriginal culture)	Factor Loading — Factor 1 racial identity	Item variance explained by factors one and two
Q2. How important is it for you to know about how Aboriginal people lived in the old days?	0.591		0.356
Q4. How important is it that you to do activities for Aboriginal Week?	0.667		0.383
Q6. How important is it for you to play with Aboriginal kids?		0.743	0.552
Q8. How important is it for you to make Aboriginal foods like damper?	0.751		0.468
Q10. How important is it for you that you’re Aboriginal?		0.446	0.262
Q12. How important is it for you to be the same as other Aboriginal kids?		0.63	0.463
Q14. How important is it for you to know about Aboriginal stories of the Dreaming?	0.551		0.515
Q16. How important is it for you to feel part of the Aboriginal group at school?		0.787	0.488
Q18. How important is it for you to talk Aboriginal words?	0.723		0.462
Q20. How important is it for you that your family tells you about being Aboriginal?	0.396		0.462
Q24. How important is it for you to have Aboriginal people as friends?		0.871	0.678
Q29. How important is it for you to be proud of being Aboriginal?		0.62	0.454

### Confirmatory factor analysis

Each model fitted has been fitted on complete (non-missing) data. Model specification was undertaken with reference to the theoretical and practical rationales for their inclusion in the design of the IRISE_C. In this sense, all models fitted here have been specified a priori.

Congeneric models were specified for each set of items and polychoric correlations along with their respective asymptotic covariance matrix were input to LISREL 9.1 and estimated via diagonally weighted least squares (DWLS). All models were identified using the procedure outlined by Joreskog and Sorbom [23]. The distributions of item data from the IRISE_C show the majority of the items to be ordinal and with markedly non-normal distributions.

The final choice of model fit indices took into account the following properties of the data: 1) a relatively simple one-factor congeneric model with uncorrelated error; 2) a small sample (N < 250); 3) item distributions that violate assumptions of normality by a high degree; and 4) a decision to use DWLS as the estimator. In line with Hu and Bentler [24] the principal model fit index was the Standardized Root Mean Residual (SRMR). This index is most sensitive to model misspecification in simple models (as opposed to misspecification in complex models). The SRMR was used in conjunction with the Non-Normed Fit Index (NNFI) to prevent estimation bias in the SRMR associated with smaller sample sizes [25]. Models were deemed to have a good fit where the SRMR < 0.05 and the NNFI > 0.95 and an acceptable fit where the SRMR < 0.10 and the NNFI > 0.90.

The final model for knowledge about Aboriginal culture was good (SRMR = 0.0434; NNFI = 1.000), and item loadings ranged from 0.51 to 0.81. The item loadings between each variable and the underlying latent factor can be interpreted as a correlation, for example for every standard deviation change in the underlying construct of ‘Knowledge of Aboriginal Culture’ we expect a 0.57 of a standard deviation change in item Q1, ‘How much do you know about how Aboriginal people lived in the old days?’ The square of the item loading represents the proportion of variance in the individual item that is explained by the underlying factor; in this case, Knowledge of Aboriginal Culture explains 32.5 % (0.57^2^) of variance in Q1 (Table 4).

**Table 4:** Confirmatory factor analysis knowledge – Aboriginal culture

*Knowledge – Aboriginal culture*	Item loadings^a^ λ_x_	Model characteristics
Q1. How much do you know about how Aboriginal people lived in the old days?	0.57	*N* = 195^b^
		df = 27^c^
Q3. How much do you know about Aboriginal Week activities?	0.51	*χ* ^2^ = 27.15^d^
Q7. How much have you learned to make Aboriginal foods like damper?	0.74	SRMR^e^ = 0.0434
		NNFI^f^ = 1.00
Q13. How much do you know about Aboriginal stories of the Dreaming (Dreamtime)?	0.65	Good
Q17. How much do you talk Aboriginal words?	0.67	
Q19. How much does your family tell you about being Aboriginal?	0.69	
Q35. How much do you go bush with your family?	0.67	
Q37. How much have you eaten Aboriginal foods, like kangaroo?	0.79	
Q39. How much do you know about the dances Aboriginal people did in the old days?	0.81	

a. Partial regression coefficients of the item on the underlying construct

^b^N = Analytic sample size

^c^df = Degrees of freedom

^d^ *χ*2 = Chi square

^e^SRMR = Standardized Root Mean Residual

^f^NNFI = Non-Normed Fit Index

The final model for knowledge about racial identity was acceptable (SRMR = 0.0598; NNFI = 0.987), and item loadings ranged from 0.57 to 0.91 (Table 5).

**Table 5:** Confirmatory factor analysis knowledge – racial identity

*Knowledge – racial identity*	Item loadings^a^ λ_x_	Model characteristics
Q5. How much do you like playing with Aboriginal kids?	0.72	*N* = 200
		df = 20
Q9. How much do you like being Aboriginal?	0.80	*χ* ^2^ = 34.64
Q11. How much are you the same as other Aboriginal kids?	0.57	SRMR = 0.0598
		NNFI = 0.987
Q15. How much do Aboriginal kids make you feel part of their group at school?	0.68	Acceptable
Q23. How much do you like Aboriginal people as friends?	0.71	
Q28. How much are you proud of being Aboriginal?	0.91	
Q30. How much do Aboriginal kids help each other?	0.66	
Q33. How much do you like the Aboriginal flag?	0.82	

^a^Partial regression coefficients of the item on the underlying construct

The final model for the salience of about Aboriginal culture was acceptable (SRMR = 0.0613; NNFI = 0.985), and item loadings ranged from 0.62 to 0.82 (Table 6).

**Table 6:** Confirmatory factor analysis salience – Aboriginal culture

*Salience – Aboriginal culture*	Item loadings^a^ λ_x_	Model characteristics
Q2. How important is it for you to know about how Aboriginal people lived in the old days?	0.62	*N* = 188
		df = 27
Q4. How important is it that you to do activities for Aboriginal Week?	0.64	*χ* ^2^ = 46.15
Q8. How important is it for you to make Aboriginal foods like damper?	0.78	SRMR = 0.0613
		NNFI = 0.985
Q14. How important is it for you to know about Aboriginal stories of the Dreaming?	0.78	Acceptable
Q18. How important is it for you to talk Aboriginal words?	0.73	
Q20. How important is it for you that your family tells you about being Aboriginal?	0.70	
Q36. How important is it for you to go bush with your family?	0.66	
Q38. How important is it for you to eat Aboriginal foods?	0.68	
Q40. How important is it for you to know about the dances Aboriginal people did in the old days?	0.82	

^a^Partial regression coefficients of the item on the underlying construct

The final model for knowledge about Aboriginal culture was good (SRMR = 0.0434; NNFI = 1.00), and item loadings ranged from 0.51 to 0.81 (Table 7).

**Table 7:** Confirmatory factor analysis salience – racial identity

*Salience - racial identity*	Item loadings^a^ λ_x_	Model characteristics
Q6. How important is it for you to play with Aboriginal kids?	0.78	*N* = 191
		df = 20
Q10. How important is it for you that you’re Aboriginal?	0.71	*χ* ^2^ = 21.10
Q12. How important is it for you to be the same as other Aboriginal kids?	0.72	SRMR = 0.0557
		NNFI = 0.999
Q16. How important is it for you to feel part of the Aboriginal group at school?	0.78	Acceptable
Q24. How important is it for you to have Aboriginal people as friends?	0.86	
Q29. How important is it for you to be proud of being Aboriginal?	0.78	
Q31. How important is it that Aboriginal kids help each other?	0.68	
Q34. How important is it for you to like the Aboriginal flag?	0.74	

^a^Partial regression coefficients of the item on the underlying construct

### Scoring scales

While the Confirmatory Factor Analysis provides a method for assessing the relationship between assessing the relationship between items and the underlying constructs for our study sample, a simple and more practical way of scoring is to create unweighted scores within each of the 4 scales.

The scoring for these scales is as follows:

#### Scale 1: Knowledge of Aboriginal culture

This scale comprised of the following 9 items:

- Q1. How much do you know about how Aboriginal people lived in the old days? +
- Q3. How much do you know about Aboriginal Week activities? +
- Q7. How much have you learned to make Aboriginal foods like damper? +
- Q13. How much do you know about Aboriginal stories of the Dreaming (Dreamtime)? +
- Q17. How much do you talk Aboriginal words? +
- Q19. How much does your family tell you about being Aboriginal? +
- Q35. How much do you go bush with your family? +
- Q37. How much have you eaten Aboriginal foods, like kangaroo? +
- Q39. How much do you know about the dances Aboriginal people did in the old days?

Ninety four percent (94.8 %) of children responded to 7 or more out of the 9 questions. The proposed usage is that for children with 7 or more responses add up all scores and then divide by number of valid responses (i.e. 9 to 36 divided by 8, or 8 to 32 divided by 8, or 7 to 28 divided by 7). This gives a score from 1–4, which can be interpreted on original scale (1 = none, 2 = − a little bit, 3 = some, and 4 = a lot).

#### Scale 2: Knowledge of racial identity

This scale comprised of the following 8 items:

- Q5. How much do you like playing with Aboriginal kids? +
- Q9. How much do you like being Aboriginal? +
- Q11. How much are you the same as other Aboriginal kids? +
- Q15. How much do Aboriginal kids make you feel part of their group at school? +
- Q23. How much do you like Aboriginal people as friends? +
- Q28. How much are you proud of being Aboriginal? +
- Q30. How much do Aboriginal kids help each other?+
- Q33. How much do you like the Aboriginal flag?

Ninety five percent (95.6 %) of children responded to 6 or more out of the 8 questions. The proposed usage is that for children with 6 or more responses add up all scores and then divide by number of valid responses (i.e. 8 to 32 divided by 8, or 7 to 28 divided by 7, or 6 to 24 divided by 6).

#### Scale 3: Salience of Aboriginal culture

This scale consisted of the following 9 items:

- Q2. How important is it for you to know about how Aboriginal people lived in the old days? +
- Q4. How important is it that you to do activities for Aboriginal Week? +
- Q8. How important is it for you to make Aboriginal foods like damper? +
- Q14. How important is it for you to know about Aboriginal stories of the Dreaming?+
- Q18. How important is it for you to talk Aboriginal words? +
- Q20. How important is it for you that your family tells you about being Aboriginal? +
- Q36. How important is it for you to go bush with your family? +
- Q38. How important is it for you to eat Aboriginal foods? +
- Q40. How important is it for you to know about the dances Aboriginal people did in the old days?

Ninety three percent (93.0 %) of children responded to 7 or more out of the 9 questions. The proposed usage is that for children with 7 or more responses add up all scores and then divide by number of valid responses (i.e. 9 to 36 divided by 8, or 8 to 32 divided by 8, or 7 to 28 divided by 7).

#### Scale 4: Salience of Racial Identity

The final scale consisted of 8 items including:

- Q6. How important is it for you to play with Aboriginal kids? +
- Q10. How important is it for you that you’re Aboriginal? +
- Q12. How important is it for you to be the same as other Aboriginal kids? +
- Q16. How important is it for you to feel part of the Aboriginal group at school? +
- Q24. How important is it for you to have Aboriginal people as friends? +
- Q29. How important is it for you to be proud of being Aboriginal? +
- Q31. How important is it that Aboriginal kids help each other? +
- Q34. How important is it for you to like the Aboriginal flag? +

Ninety two percent (92.1 %) of children responded to 6 or more out of the 8 questions. The proposed usage is that for children with 6 or more responses add up all scores and then divide by number of valid responses (i.e. 8 to 32 divided by 8, or 7 to 28 divided by 7, or 6 to 24 divided by 6).

The characteristics of the 4 scales are described in Table 8 below. Although the scales were developed based on a hypothesis of the relationship between racial self-esteem and overall wellbeing, this study has only assessed the internal consistency of these scales, not the relationship between these scales and external measures of wellbeing. Future research however, will be conducted to test the relationship between the IRISE scales and generic measures of wellbeing in due time. Thus, interpretation of cut-points in the current scales needs to made with caution. The median of the 4 scales at the 25th percentile is 3.00, thus a simple interpretation which could be made for a child scoring 3 or less on any of the scales is that that child sits in the lowest quartile for that scale.

**Table 8:** Scored scales

		Knowledge of Aboriginal culture	Knowledge of racial identity	Salience of Aboriginal culture	Salience of racial identity
N	Valid	217	219	213	211
	Missing	12	10	16	18
Mean		2.98	3.51	3.28	3.46
Median		3.11	3.62	3.44	3.62
Mode		3.44	4.00	4.00	4.00
Std. Deviation		.68	.50	.65	.590
Variance		.47	.25	.42	.349
Range		2.89	2.50	2.89	2.88
Minimum		1.11	1.50	1.11	1.13
Maximum		4.00	4.00	4.00	4.00
Percentiles	5	1.67	2.43	1.86	2.12
	10	2.00	2.88	2.38	2.75
	15	2.22	3.00	2.62	2.88
	20	2.33	3.25	2.89	3.00
	25	2.44	3.25	2.89	3.12
	33	2.67	3.43	3.07	3.37
	50	3.11	3.62	3.44	3.62
	66	3.44	3.75	3.67	3.88
	75	3.56	3.88	3.78	3.87
	80	3.64	3.88	3.89	4.00
	85	3.67	4.00	3.89	4.00
	90	3.78	4.00	4.00	4.00
	95	4.00	4.00	4.00	4.00

Similarly, the median of the 4 sub-scales at the 75th percentile is 3.82. Thus, any child scoring 3.82 or above any given scale could be interpreted as sitting in the top quartile for that scale.

Table 9 below shows that the correlations between the 4 final scales suggest an inter-relationship between the 4 scales.

**Table 9:** Correlations between the 4 scales

		Knowledge of Aboriginal culture	Knowledge of racial identity	Salience of Aboriginal culture	Salience of racial identity
Knowledge of Aboriginal Culture	Pearson Correlation	1	.565^a^	.709^a^	.526^a^
	Sig. (2-tailed)		.000	.000	.000
	N	217	217	213	210
Knowledge of Racial Identity	Pearson Correlation	.565^a^	1	.550^a^	.784^a^
	Sig. (2-tailed)	.000		.000	.000
	N	217	219	213	211
Salience of Aboriginal Culture	Pearson Correlation	.709^a^	.550^a^	1	.633^a^
	Sig. (2-tailed)	.000	.000		.000
	N	213	213	213	210
Salience of Racial Identity	Pearson Correlation	.526^a^	.784^a^	.633^a^	1
	Sig. (2-tailed)	.000	.000	.000	
	N	210	211	210	211

^a^Correlation is significant at the 0.01 level (2-tailed)

## Discussion

The IRISE_C explored the identity and related self-esteem for 8–12 year-old Australian Aboriginal children. It was developed by the first author, an Aboriginal researcher. A series of consultations, negotiations and reviews from other Aboriginal community members, Aboriginal teachers, and professionals ensured that the concepts contained in the IRISE_C were culturally sound and acceptable. Furthermore, the recruitment of Aboriginal Community Research Assistants who reside in the local research sites have also contributed to acknowledging and following Aboriginal ways of working. In following such a protocol, a high level of Aboriginal ownership has been encouraged and in doing so, the development of the IRISE_C inventory and culturally safe and secure procedures have ensured authentic and valid results. Further, the concepts captured by the instrument have been deemed of value and acceptability by the Aboriginal carers who provided a high response rate of consent for their children to participate in the study.

Statistically, this study has demonstrated that the IRISE_C is a valid and reliable instrument that captures identity and self-esteem for Australian Aboriginal children 8–12 years of age. The confirmatory factor analysis has shown that the 4 subscales: 1. knowledge of identity, 2. salience of identity, 3. knowledge of culture and 4. salience of culture represent “good” and “acceptable” fitting models. This demonstrates that each sub-scale effectively captures a single, consistent underlying factor or concept. Further, the structure of domains identified through the factor analytic approach matches the domains identified through the consultative processes as reflecting the underlying constructs of Aboriginal racial identity. As the items for each sub-scale have been developed through a multi-stage, iterative consultative process with the local community, it is likely that the concepts being assessed by each scale have meaning to the families in the communities in which the scale has been tested.

This study does have some limitations. Results may not be generalised to Aboriginal children living in remote areas as these children were not included in the study. Furthermore, the current sample size for urban, regional and rural sites may not have been large enough to adequately describe the full diversity of identity and self-esteem of Aboriginal children. In Western Australia, there are approximately 69 665 [26] Aboriginal people however, of these numbers there are more than 250 Aboriginal communities (not including Aboriginal residents in metropolitan areas) [27]. Within these communities are distinct Aboriginal groups with a complex and rich system of language groups and skin groups- all of which raise claim to an Aboriginal identity. In comparison, only 8 Aboriginal groups were identified and most children identified as Noongar (from the south-west of Western Australia). It is anticipated that a difference in the concepts contained in the IRISE_C may be different for other Aboriginal groups not yet researched. Another challenge is the influence of dual or multi-identities children may have of their Aboriginal identity and this may include identity with more than one Aboriginal group but also with non-Aboriginal heritage.

Lastly, the IRISE_C provides a snap-shot of identity and self-esteem at one time point. It is a static measure of identity and self-esteem however according to the current literature, identity is dynamic and self-esteem is influenced by significant others.

## Conclusion

We know very little about what protects or dismantles Aboriginal identity when other identities are present. We also are unaware of the rules, protocols, values and practices children engage in to identify in the first place. What interaction do children have with their carers/kin/parents in determining identity? Who else plays a significant role and how is identity transmitted? Furthermore, since identity is dynamic, then the feelings children experience daily need to be taken into account on the day of data collection. Future research therefore, needs to take into account the environment, setting (community), cultural protocols, significant others and the dynamic nature of identity and related self-esteem. Hence, a longitudinal study is warranted that explores the growth of identity and self-esteem over time and in particular settings and environments. In this way, we can truly understand what protects and harms the identity and related self-esteem of Australian Aboriginal children over time.

Importantly, the methods used in this study have ensured Aboriginal participants and their families were not just spectators or ‘a part’ of the research process. Aboriginal participants, their families, Aboriginal reviewers and Aboriginal community research assistants were the hub of the research wheel and who determined how fast and which direction to proceed. More specifically, they were integral from the inception to dissemination and translation of the research process. The outcome is an authentic and culturally responsive instrument that has scientific validation but importantly it has cultural validation and acceptance from the Aboriginal community. Aboriginal cultural knowledge combined with Aboriginal ownership using culturally secure methods will result in authentic and sustainable outcomes when Aboriginal research is in the hands of Aboriginal people…this is the direction that future research needs to journey.
